# Short-Term In Vitro Culture of Human Ovarian Tissue: A Comparative Study of Serum Supplementation for Primordial Follicle Survival

**DOI:** 10.3390/life15101509

**Published:** 2025-09-25

**Authors:** Serena Marcozzi, Rossella Vicenti, Gina La Sala, Harpreet Kaur Lamsira, Catello Scarica, Nicole Bertani, Massimo De Felici, Raffaella Fabbri, Francesca Gioia Klinger

**Affiliations:** 1 Section of Histology and Embryology, Department of Biomedicine and Prevention, University of Rome Tor Vergata, 00133 Rome, Italy; 2 Division of Gynaecology and Human Reproduction Physiopathology, IRCCS Azienda Ospedaliera-Universitaria di Bologna, 40100 Bologna, Italy; 3 Institute of Biochemistry and Cell Biology, Italian National Research Council (CNR), 00015 Monterotondo Scalo, Italy; 4 New Fertility Group Centre for Reproductive Medicine, European Hospital, 00100 Rome, Italy; 5 Saint Camillus International University of Health Sciences, 00131 Rome, Italy

**Keywords:** fertility preservation, ovarian follicles, ovarian cortical tissue, in vitro culture, medium supplements

## Abstract

Optimizing in vitro culture conditions for cryopreserved–thawed human ovarian cortical fragments (OCFs) represents a critical step in fertility preservation strategies. OCFs predominantly contain primordial follicles (PMFs), whose survival and integrity are essential for ex vivo folliculogenesis. This study aimed to evaluate the impact of different culture media supplementations on PMF survival and tissue morphology by comparing alpha-Minimum Essential Medium (αMEM) supplemented with Human Serum Albumin (HSA), Human Serum (HS), or Serum Substitute Supplement (SSS). Twenty-nine OCFs were cultured for three days, and follicular density and were morphology assessed. Generalized linear mixed model analysis showed that PMF density was significantly higher in OCFs cultured in medium supplemented with SSS (213 PMFs/mm^3^) compared to those cultured with HSA (107 PMFs/mm^3^) or HS (93 PMFs/mm^3^). Furthermore, SSS supplementation was associated with a significant increase in the number of PMFs showing healthy morphologies. These findings indicate that SSS supplementation to αMEM enhances the survival and preserves better morphologies of the human PMFs in short-term culture, highlighting its potential as a suitable culture supplement for ovarian tissue preservation.

## 1. Introduction

A large number of young women of reproductive age are diagnosed with cancer each year. According to the latest data from the International Agency for Research on Cancer (IARC), approximately 9,6 million women worldwide are currently living with a cancer diagnosis and about 20% of them are under the age of 45 [1,2].

Recent advances in cancer diagnosis and the introduction of new chemo-radiotherapy protocols have significantly increased the survival rates in both children and young women with cancer [3]. Among these protocols, particularly those alkylating agents, such as cyclophosphamide, have the potential to be gonadotoxic with a significant impact on reproductive potential [4]. In women, this gonadotoxicity is often associated with follicular depletion and can lead to primary ovarian insufficiency (POI) [5].

Although embryo or oocyte cryopreservation is generally considered the standard protocol for fertility preservation [6], it is not feasible for pre-pubertal girls, and it may not be appropriate for women with cancer due to individual conditions and treatment schedules. For these reasons, in recent years the area of fertility preservation has seen a rapid evolvement of techniques [7].

Ovarian tissue cryopreservation and transplantation (OTC-T) is a promising method for fertility preservation in both pre-pubertal girls and adult patients with high risk of POI. Slow freezing is currently the established method for OCT, while vitrification is still being investigated as a promising alternative technique [8,9,10,11]. Cryopreservation preserves hundreds of primordial follicles (PMFs) that can be transplanted simultaneously, and it is a valid strategy to preserve endocrine function. Previous studies have reported pregnancies and live births after transplantation of cryopreserved and thawed ovarian tissue, demonstrating that OTC-T is a safe alternative method for fertility preservation [6,12,13,14,15].

However, the success of OTC-T still has multiple challenges, such as the low number of follicles in the graft, that may affect their longevity as well as the survival of the tissue during ex vivo processing and subsequent transplantation. In addition, ovarian tissue transplantation increases the risk of cancer cells recurring in patients with neuroblastoma, acute lymphoblastic leukemia and ovarian cancer [16,17,18,19]. With the aim to avoid the potential risk of reintroducing malignant cells, in vitro growth (IVG) of early-stage follicles combined with in vitro maturation (IVM) of the resulting oocytes has been explored as a potential alternative approach for fertility preservation, to generate developmentally competent oocytes entirely ex vivo. Recent studies have highlighted the feasibility of IVG–IVM protocols and the contribution of culture additives to improve follicle survival and growth in vitro [20,21]. Nevertheless, IVG and IVM remain largely experimental, and their clinical application is still limited [22,23]. For such reasons, further optimization of culture conditions to preserve follicle morphology and survival remains a key objective in fertility preservation research [24].

Since the first culture in the 1990s [25], various methods for culturing human ovarian cortical tissue have been developed considering different parameters such as media volume, media type, and supporting tissue membranes [26]. However, the complexity of ovarian folliculogenesis, which relies on tightly regulated endocrine, paracrine, and autocrine pathways [27], has limited the results obtained so far. Therefore, the optimization of ovarian tissue culture to increase the survival and development of healthy multilayer follicles is of paramount importance for the realization and subsequent clinical translation of this approach [26].

To date, a standardized or optimized culture system for the in vitro development of human ovarian follicles remains elusive. The key challenge for the prospective clinical application of this method is the identification of an optimal culture system for human ovarian tissue that will be aimed at maximizing the yield of healthy multilayer follicles [26].

According to the literature, the most commonly used medium for the in vitro culture of human ovarian cortical tissue is a culture medium supplemented with human serum albumin (HSA), commonly used as a carrier and antioxidant support in culture media. This supplementation has been shown to enhance both follicle recovery and subsequent growth, following the thawing of cryopreserved ovarian tissue, highlighting its beneficial role in tissue viability and function [26,27,28,29,30,31,32,33]. In our laboratory, it is a standard clinical and research practice to supplement the culture medium with human serum (HS), a more complex and heterogeneous biological fluid, containing albumin, globulins, growth factors, and other proteins, but it is subject to donor variability. This approach has consistently yielded satisfactory results, particularly in terms of follicular recovery, viability, and overall quality of the tissues [34,35,36,37]. Nevertheless, we sought to determine whether follicle recovery and their maintenance in culture over a few days could be further improved. SSS (Serum Substitute Supplement) has been developed to provide a stable and defined alternative to human serum in assisted reproduction. It contains purified human albumin combined with a defined mixture of α- and β-globulins, minimizing batch-to-batch variability. In the context of In Vitro Fertilization (IVF) embryo culture system, SSS has been shown to support fertilization, blastocyst formation, and reduce oxidative stress [38,39,40].

Based on these properties, we hypothesized that SSS supplementation could better preserve the morphology and survival of primordial follicle during short-term culture of cryopreserved human ovarian cortical tissue, compared to HS and HSA. To this aim, human ovarian cortical fragments (OCFs) were cultured for three days in α-MEM supplemented with either HS, HSA or SSS. At the end of the culture, OCFs were subjected to histological analysis to assess follicle classification, density, and morphological integrity of granulosa cells and oocytes. The resulting data were subjected to statistical evaluation using a generalized linear mixed model (GLMM), allowing correction for intra-sample variability and repeated measurements.

## 2. Materials and Methods

### 2.1. Patient History

Ovarian cortical tissue samples were collected by laparoscopy from three patients aged 14, 15, and 25 years, affected by non-gynecological neoplasms, who had cryopreserved their ovarian tissue before undergoing anticancer treatment at the Division of Gynaecology and Human Reproduction Physiopathology of IRCCS Azienda Ospedaliero-Universitaria of Bologna, Italy (Table 1). The selected age range reflects the typical profile of patients undergoing fertility preservation in our clinical setting, whose ovarian tissue typically exhibits a higher density of primordial follicles, thereby offering optimal conditions for in vitro studies. The informed consent was obtained from all the patients, and the study was approved by the Ethics Committee of the Policlinico S.Orsola-Malpighi (protocol 482/2018/Sper/AOUBo). Cryopreserved ovarian tissue transplantation was not performed and all collected tissue was donated in its entirety for research purposes.

### 2.2. Ovarian Tissue Collection, Freezing, and Thawing

Ovarian biopsies were collected during surgery and immediately transferred to the laboratory in Dulbecco’s phosphate-buffered saline (DPBS; Gibco, Life Technologies, Paisley, Scotland) supplemented with 10% HS at 4 °C according to Fabbri et al. 2012 [34]. The ovarian medulla was removed using surgical scissors and the ovarian cortical tissue was dissected into strips (±15 mm × 2 mm × 1 mm), then subjected to slow freezing. HS was obtained from the Transfusion Center of S. Orsola-Malpighi Hospital as follows: whole blood was collected from different female donors who fulfilled all standard eligibility criteria for blood donation, including negative infectious tests, normal blood count, normal ECG, female sex, fertile age, absence of ongoing estrogen–progestin therapy. Blood samples were allowed to clot by leaving it to stand undisturbed at room temperature for 15–30 min. The clot was removed by centrifugation at 1000–2000× *g* for 30 min in a refrigerated centrifuge, and the resulting supernatant serum was collected. Serum was then heat-inactivated at 56 °C for 1 h immediately after collection, pooled, and stored at −20 °C until use. The same stored serum was used in the experiments described.

A slow freezing protocol was used according to Fabbri et al., 2010 [41]. Ovarian cortical strips were placed in pre-cooled plastic cryovials (Intermed Nunc Cryotubes, Roskilde, Denmark) containing 1.5 M 1,2-propanediol (PROH—Fluka Chemica, Sigma Aldrich SrL; Milan, Italy), 0.2 M sucrose (Fluka Chemica, Sigma Aldrich SrL; Milan, Italy), and 30% HS in DPBS and maintained at 0 °C in an ice bath. The cryovials were transferred to a rolling system (Continents instrument, Amityville, NY, USA) for 30 min at 4 °C; they were then cooled in a programmable freezer (Planer Kryo 10/1.7 Series III, SAPIO Life, Milan, Italy) allowing the gradual reduction in the temperature from 0 to −140 °C. After 10 min of temperature stabilization, the cryovials were transferred into liquid nitrogen tanks and stored until thawing. Immediately before the culture experiments described below, the ovarian cortical tissues were rapidly thawed as previously described [35]; at least three ovarian cortical tissues were used from each patient. Briefly, the cryovials were air-warmed for 30 s and then immersed into 37 °C water bath for 2 min. The cryoprotectants were removed at 4 °C by four-stepwise dilution: (i) 0.76 M PROH, 0.175 M sucrose, 30% HS in DPBS for 5 min; (ii) 0.26 M PROH, 0.175 M sucrose, 30% HS in DPBS for an additional 5 min; (iii) 0.175 M sucrose, 30% HS in DPBS for 10 min and finally; (iv) DPBS supplemented with 30% HS for 20 min.

### 2.3. Media Preparation

Basic culture medium (BCM) consisted of alpha minimal essential medium (αMEM, Aurogene, Rome, Italy) supplemented with 2 mM L-glutamine, antibiotics (50 U/mL penicillin G, 50 μg/mL streptomycin), 25 mM N-acetyl-L-cysteine, and 1% ITS liquid media supplement (all from Sigma-Aldrich, Milan, Italy). BCM was further supplemented with 5 mg/mL human serum albumin (HSA, FUJIFILM Irvine Scientific, Santa Ana, CA, USA), or with 40% human serum (HS), according to protocols from Fabbri and colleagues [34,35,36,37], or 6 mg/mL serum substitute supplement (SSS, FUJIFILM Irvine Scientific, USA). SSS consists of 84% human serum albumin from therapeutic-grade source material (50 mg/mL, 5% *w*/*v*) and 16% human serum globulins (10 mg/mL, 1% *w*/*v*) in a saline solution. From here onwards, the three experimental conditions will be referred to as HSA, HS, and SSS.

### 2.4. Ovarian Tissue Culture

**Figure 1:**
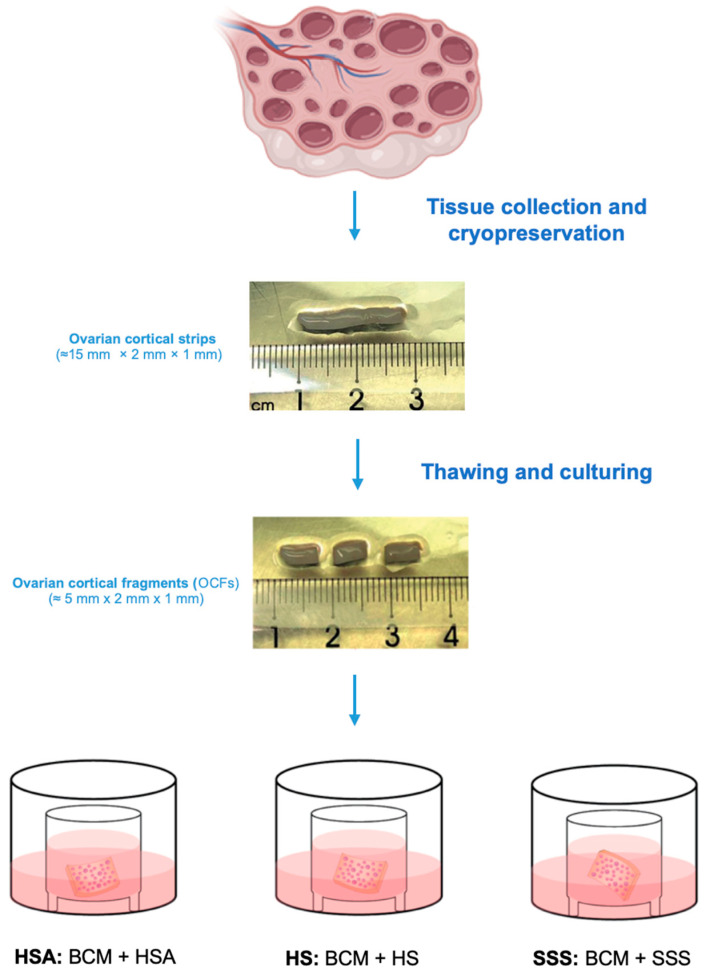
Ovarian cortical tissues were collected, dissected into strips (±15 mm × 2 mm × 1 mm), and cryopreserved. After thawing, the cortical strips were cut into smaller OCFs (±5 mm × 2 mm × 1 mm) and cultured for three days under different conditions. OCFs were placed on Millicell culture plate inserts and maintained in BCM. Three different culture conditions were tested. HSA group: BCM + 5 mg/mL HSA; HS group: BCM + 40% HS; SSS group: BCM + 6 mg/mL SSS. Each experimental condition was independently replicated three times.

Each ovarian cortical tissue was sectioned into smaller OCFs (±5 mm × 2 mm × 1 mm) using a scalpel under sterile conditions, in an open-air setting in PBS. OCFs were placed onto Millicell-CM culture plate inserts (12 mm outer diameter, 10 mm inner diameter, 0.4 μm pore size, PIHA01250, Millipore, Sigma Aldrich SrL; Milan, Italy) within separate wells of a 24-well culture plate (Corning, Turin, Italy). Two OCFs were seeded for insert. Before the addition of OCFs, 200 μL of culture medium was added to the insert and an additional 400 μL to the surrounding well. OCFs from each patient were randomly assigned to the different experimental treatments and cultured for three days at 37 °C in 5% CO_2_, in a 95% humidified incubator in BCM to which the three different additives, HSA, HS or SSS, were added. Given that ovarian tissue transplantation protocols often involve a short culture period following thawing, a three-day duration used in this study was selected to standardize experimental conditions and provide an experimental model to assess early follicular responses. Medium was changed after 48 h. The experimental workflow for tissue collection and culture is depicted in Figure 1. OCFs from each patient were randomly distributed among the three different culture conditions. From each patient, 9 to 10 OCFs were obtained and randomly distributed among the three experimental culture conditions, with 8 to 11 OCFs assigned to each culture condition, ensuring that 3 to 4 OCFs from each patient were included in every group. This randomization aimed to minimize variability and ensure reliable comparison between the experimental groups.

### 2.5. Histological Examination

At the end of the culture period, OCFs were fixed in 4% formalin solution at room temperature for 5 h and then kept at 4 °C ON. After alcohol dehydration in ascending concentrations of ethanol, samples were embedded in paraffin wax and serial sections of 5 μm thickness were cut following standard histological procedures. After deparaffination and hydration, the sections were stained with haematoxylin and eosin for morphological assessment. Follicle counts were performed every six sections, over the entire sample. The developmental stages of follicles were assessed according to Gougeon classification [42], based on the shape and number of granulosa cells’ (GCs) layers surrounding the oocytes. Primordial follicles (PMFs) were classified as those with a single layer of flattened GCs, primary follicles (PFs) with a single layer of one or more cuboidal GCs, and growing follicles (GFs) where at least part of the follicle had two layers of cuboidal GCs. Follicles were considered healthy when they presented an intact oocyte surrounded by a uniform layer of flattened GCs. On the other hand, follicles were classified as unhealthy when they exhibited at least one of the following features: oocyte or GCs with condensed chromatin, shrunken or vacuolated ooplasm, GC layers detached from the oocyte or a combination of both oocyte and GCs alteration. To avoid double counting, only oocytes with a visible nucleus were considered in the analysis. Follicles classification and counts were performed by two independent researchers [28,30,31]. Images were taken under a Zeiss Axioplan 2 microscope equipped with NIS-Elements F3.22.00 (Build 710) as acquisition software. A digital image analysis system (Image J, software version 1.53k; NIH, Image SXM) was used to measure the area of each section studied. The total volume of OCF, expressed in mm^3^, was calculated by the sum of the measured area (mm^2^) across all analyzed sections, multiplied by 6 (the interval between sections analyzed) and then by 0.005 mm (section thickness). Follicle density was calculated considering only morphologically healthy follicles and by dividing the total number of follicles counted in the sample by the volume (V) of the analyzed OCF (i.e., PMFs number/V mm^3^). The percentage of healthy follicles at each developmental stage was calculated based on the total number of follicles counted. An additional count was performed to assess, within each follicle class, the distribution of healthy versus unhealthy or altered follicles. All data are presented as mean ± standard error of the mean (SEM).

### 2.6. Statistical Analysis

All analyses were performed using Stata 18 (StataCorp, College Station, TX, USA). Proportions of follicle stages within each patient sample were compared to calculate overall means and SEM. We employed a generalized linear mixed model (GLMM) with a negative binomial distribution to account for overdispersion and within-sample correlation as multiple observations were obtained from the same ovarian fragment. The GLMM included treatment type (HSA, HS, SSS), follicle type (PF, PMF), and patient age as fixed effects, while OCF was modeled as a random effect to correct for repeated measurements within the same sample. A log-link function was applied, and statistical significance was assessed using a likelihood ratio test. To assess difference in follicle morphology and binary outcome (e.g., normal vs. degenerated) a logistic regression model was used. The model allowed to evaluate the impact of culture treatments (HSA, HS, SSS), adjusted for follicle type and patient. Statistical significance was assessed using the Wald chi-squared test, with a significance level set at *p* < 0.05. Results are presented as mean ± SEM.

## 3. Results

To evaluate the impact of different culture additives on follicular preservation, OCFs from three patients with different oncological conditions (Table 1) were cultured for three days in the presence of HSA, HS, or SSS, as illustrated in Figure 1. At the end of the culture period, follicles were classified and quantified based on established morphological criteria into primordial (PMFs), primary (PFs), and growing follicles (GFs) (Table 1).

**Figure 2:**
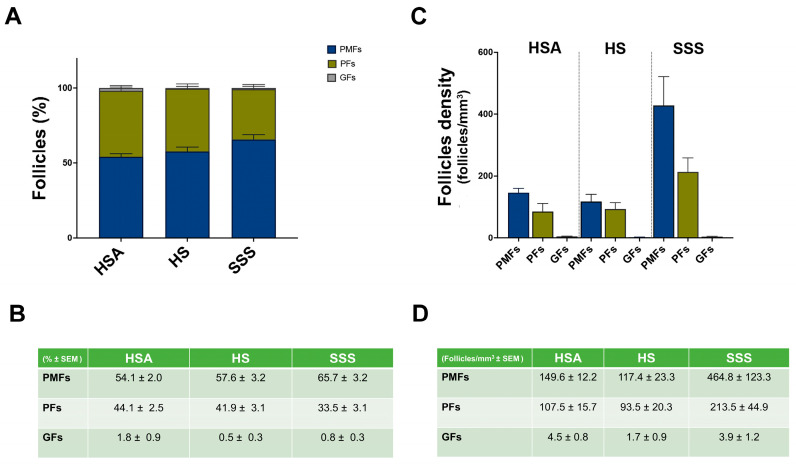
Follicle count and density analysis across different culture conditions. (**A**) Percentage distribution of PMFs, PMs, and GFs observed in OCF cultured under HSA, HS, and SSS conditions. Follicles were classified as PMFs, PFs, and GFs as described in Materials and Methods. Data are presented as the mean percentage ± SEM of follicles out of a total 65,986 counted follicles from three patients. (**B**) Table reporting the value of the mean percentage of follicles ± SEM of each follicle type corresponding to panel A. (**C**) Follicle density (follicles/mm^3^) across different culture conditions, categorized by follicle type. Bars represent mean values ± SEM. (**D**) Table reporting the value of the follicles/mm^3^ ± SEM shown in panel (**C**).

In all OCFs, follicles were identified in both central and peripheral areas and, as expected, the majority were PMFs in all three culture conditions, followed by PFs, while GFs were detected only occasionally (Figure 2A,B).

A significantly higher density of PMFs was observed in OCFs cultured in SSS compared to those cultured with HSA or HS; in contrast, no differences were observed in the density of PFs or GFs across the treatment groups (Figure 2C,D).

To further assess these findings, GLMM with a negative binomial distribution and a log-link function was used to evaluate the relationship between follicle density and key predictors, including follicle count, follicle type, treatment type, and patient age. Random intercepts were specified for each patient to account for within-subject clustering. These analyses showed that the effect of total follicle count on follicle density was minimal and not statistically significant (β = 0.00005, *p* = 0.317, 95% CI −0.000048 to 0.0001481), suggesting that the total number of follicles did not substantially influence the calculated follicle density in this model. By contrast, follicle type significantly influenced density: both PMFs (β = 4.107, 95% CI: 3.681–4.533, *p* < 0.001) and PFs (β = 3.664, 95% CI: 3.253–4.075, *p* < 0.001) were strongly associated with increased follicle density compared to the reference category. The effects of different treatments on follicle density are summarized in Table 2. Using HS as a reference, treatment with HSA (β = 0.413, 95% CI: 0.014–0.812, *p* = 0.042) or SSS (β = 0.883, 95% CI: 0.490–1.276, *p* < 0.00) was associated with higher follicle counts, with a greatest effect observed in the SSS group. Specifically, the model indicated that follicle density in SSS condition was almost 0.9 units higher than in the HS group. This effect was not only statistically significant but also greater than that observed with HSA, suggesting that SSS provides the greatest improvement in follicle density among the tested conditions.

**Figure 3:**
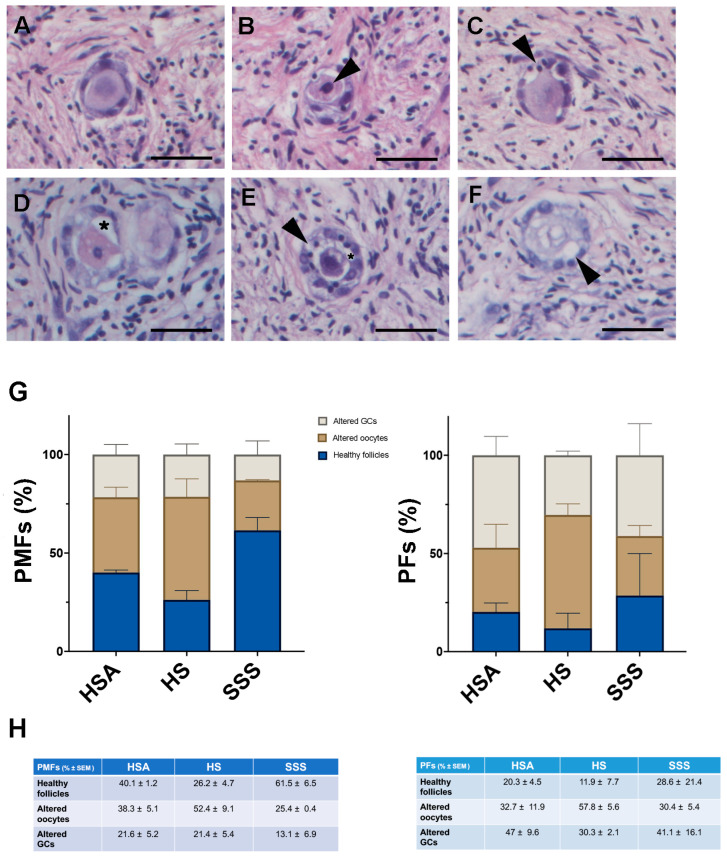
Histological assessment of follicular health across different culture conditions. (**A**–**F**) Representative images of follicular morphology. A morphologically healthy PMF is shown in (**A**). Follicles were classified as unhealthy when they exhibited oocyte ((**B**), arrowhead) or GCs ((**C**), arrowhead) with condensed chromatin, granulosa cell layers detached from the oocyte ((**D**), asterisk), or both altered oocyte and GCs ((**E**), arrowhead and asterisk) and oocyte with vacuolated ooplasm ((**F**), arrowhead). Scale bar = 25 μm. (**G**) Quantification of follicular health. Percentage of PMFs and PFs classified as morphologically healthy follicles, follicles with altered oocytes, and follicles with altered GCs. Data are presented as the mean percentage ± SEM of follicles in OCFs out of a total of 718 follicles, pooled from three patients. (**H**) Table reporting the value of the mean percentage of follicles ± SEM of each follicle type corresponding to panel (**G**).

Follicles were classified healthy (Figure 3A) or unhealthy (atretic) based on morphological criteria, including the presence of condensed chromatin in the oocytes or granulosa cells (GCs) (Figure 3B,C), detachment of GC layer/s from the oocyte (Figure 3D), a combination of these morphologies (Figure 3E) or the presence of an oocyte with shrunken or vacuolated ooplasm (Figure 3F). According to these criteria, the percentage of unhealthy PMFs was lower in SSS group compared to the other treatments (% ± SEM: HSA: 59.9 ± 1.2; HS: 73.8 ± 4.7; SSS: 38.5 ± 3.8) (Figure 3G). In contrast, no significant differences in PFs among the groups were observed (HSA: 79.7 ± 4.5; HS: 88.1 ± 7.7; SSS: 80.9 ± 15.6) (Figure 3G).

To evaluate the impact of the different additives on such morphology outcomes, a logistic regression analysis was conducted, adjusting for follicle type. The analyses revealed that compared to the reference group HSA, treatment with SSS was associated with a significantly increased odds of healthy morphology (OR = 7.81, 95% CI: 4.56–13.39, *p* < 0.001), while HS treatment was not (OR = 0.79, 95% CI: 0.51–1.21, *p* = 0.275). The coefficient for PMF type (β = 0.9880, *p* = 0.00642) indicated that PMFs had a significantly higher likelihood of retaining normal morphology compared to PFs. Furthermore, post hoc pairwise comparisons of the estimated marginal means (on the logit scale, averaged over follicle types) showed no significant difference between HSA and HS treatments (difference = 0.24, *p* = 0.8259), whereas both HSA and HS differed significantly from SSS (HSA vs. SSS: difference = −2.06, *p* < 0.0001; HS vs. SSS: difference = −2.30, *p* < 0.0001). These findings suggest that SSS treatment is associated with substantially improved preservation of follicular morphology compared to HSA and HS, while the effect of HS treatment appears negligible.

**Figure 4:**
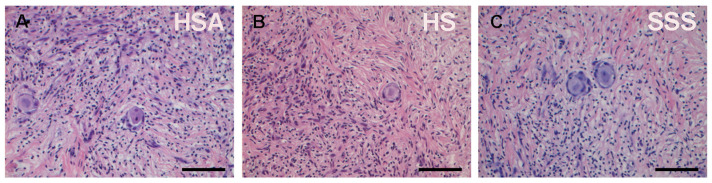
Histological assessment of OCFs across different culture conditions. (**A**–**C**) Representative images of OCFs sections stained with H&E, showing the overall stromal architecture in OCFs cultured with HSA, HS, or SSS. Scale bar = 50 μm.

Finally, the stroma appeared homogenous and compact in the OCFs of the three experimental groups with no morphological differences observed (Figure 4A–C).

## 4. Discussion

Developing optimized in vitro culture systems for human ovarian cortical tissue remains critical for fertility preservation. Several approaches have been taken to grow human follicles in vitro with the use of fresh or thawed cryopreserved ovarian cortical tissue [7,30,43,44]. Moreover, various basal media (e.g., MEM, DMEM, McCoy 5α, EBSS) and mixed formulations (e.g., DMEM/F12, α-MEM + Glutamax) have been tested, often supplemented with nutrients, hormones, or growth factors to support follicular survival and function [45,46,47,48]. It is now clear that for complete development of follicles, a multistep culture system is required to support each stage of folliculogenesis (for a review, see [49]). However, due to the predominance of quiescent primordial follicles (PMFs) in cortical tissue, their maintenance and survival represent a key challenge. A multistep culture system is considered necessary to support full folliculogenesis [49].

The present study underscores the critical role of culture media composition in preserving human PMF survival and maintaining ovarian stromal integrity, thereby contributing to the growing body of evidence supporting the need for optimized in vitro culture systems for this class of follicles which constitute the ovarian reserve. Our results demonstrate that the use of medium supplemented with SSS resulted in a significantly higher density of PMFs in OCFs and reduced unhealthy morphological features in both granulosa cells and oocyte, compared to media supplemented with HSA or HS. These effects were not observed for PFs or GFs, suggesting that SSS specifically enhance PMF maintenance in culture, which is the first step for the subsequent stages of folliculogenesis.

Our analyses demonstrate that the higher number of PMFs found in SSS, in comparison to the other additives, was independent from the initial follicular density, highlighting its advantages over HSA and HS in preserving follicular viability during short-term culture. Moreover, culturing OCFs with SSS showed advantages not only in follicle count but also in better-preserved follicular morphology, particularly for PMFs, compared to HSA and HS. SSS, derived from a purified fraction of human serum and consisting primarily of alpha- and beta-globulins and serum albumin, has been previously reported to enhance outcomes in IVF embryo culture systems [40,50,51,52,53]. Despite these promising results, SSS is not widely or consistently used in ovarian tissue culture, possibly due to the preference for traditional supplementation methods, such as HSA, which have been more extensively studied in reproductive research. The enhanced efficacy of SSS reported here could be attributed to its composition, which provides a more physiologically relevant balance of proteins and bioactive factors compared to HSA and HS. Unlike HS, which contains a broader and less controlled mixture of serum proteins, and HSA, which primarily functions as a carrier protein [54], SSS includes additional globulins that may support cell adhesion, growth, and survival [50]. This optimized formulation could create a more supportive microenvironment for follicular maintenance by reducing oxidative stress and promoting granulosa cell interactions, thereby improving follicle integrity during culture [55]. While the improved preservation seen in the SSS group could be related to its antioxidant or globulin content, this is merely speculative without direct compositional or functional analysis. Future work will explore the underlying mechanisms using oxidative stress markers and proteomic or metabolomic analyses.

As reported above, the choice of a short three-day culture period was based on the requirements of ovarian tissue thawing and transplantation techniques (for a review, see [56]). In fact, limiting the culture period to three days aligns with clinical protocols to maintain follicular viability while minimizing potential stress-induced activation of PMFs, a relevant aspect for pre-transplant culture settings, as described by Dolmans et al. (2021) [13]. Extending the culture period beyond this duration could lead to non-physiological follicular activation and loss of quiescence, which would negatively impact tissue function after transplantation [57]. Nevertheless, extending the culture duration will be essential to determine whether SSS sustains its benefits over time, a critical factor for optimizing in vitro follicle maturation (IVM) and transplantation strategies. Moreover, a deeper analysis of the culture medium through metabolomic and proteomic profiling could reveal key biomolecules and signaling pathways involved in follicular maintenance, improving the development of more refined culture conditions. Finally, investigating oxidative stress markers in SSS-treated tissues may provide insights into its potential antioxidant effects, further explaining its role in preserving follicular integrity.

A key challenge in assessing PMF survival in cultured human OCFs is the inherent variability in the follicular cohort within the ovarian cortex. The ovarian cortex exhibits heterogeneous follicle distribution and a wide variation in follicle density [32], making it difficult to compare the numbers of follicles in a piece of control starting tissue with those in pieces subjected to different treatments in vitro. Nevertheless, Schmidt et al. [32] reported a significant inverse correlation between follicle density and age across 21 randomly selected patients. To account for this variability and to overcome the small sample size in the present study due to challenges of assessing human ovarian tissue from young cancer patients, we applied a GLMM, which adjusted for intra-sample correlations and overdispersion in the data. By including OCF as a random effect, the model controlled for inherent differences between samples, ensuring that variations in follicle number were attributable to culture conditions rather than intrinsic differences in follicular distributions between fragments, thus increasing the statistical robustness of our findings. Although the number of patients was limited, the consistency of the observed effects across all samples, particularly the significantly improved primordial follicle survival in the SSS condition, supports the reliability of the data.

Our findings contribute to the ongoing efforts in optimizing short-term in vitro culture of human ovarian tissue by comparing HSA, HS, and SSS under identical culture conditions. The comparative analysis between SSS, HS, and HSA underlines the importance of supplement composition in preserving PMF morphology and density during early culture, supporting a more physiologically stable environment. This complements previous observations by Fabbri et al. (2006, 2012), who demonstrated satisfactory morphological preservation using 40% HS, but without exploring alternative formulations or comparative efficacy [12,34]. Unlike the study of Bjarkadottir et al. (2021), which focused on basal medium composition and physical culture conditions, showing that low-volume αMEM supports superior follicle morphology after 6 days, our three-day culture condition captures the initial stages of morphological preservation following thawing and aims to minimize pre-transplant stress [26]. Although Lotz et al. (2022) did not directly investigate in vitro culture systems, their findings emphasize the importance of optimizing every step of fertility preservation protocols, from tissue cryopreservation to transplantation [15]. In this context, our study contributes to improving short-term pre-transplant culture strategies, which may influence overall graft quality and clinical outcomes.

Stromal cell density is an important indicator of tissue integrity and is likely to play a role in folliculogenesis through cellular mechanosensing. Moreover, in vitro culture can cause remodeling of the ovarian ECM and contribute to PMF activation [57]. In none of the experimental culture conditions, histological evidence of stromal modification or PMF activation was observed. However, we recognize the need to integrate a more detailed analysis using molecular and functional markers, such as FOXO3A, Ki67, caspase-3, and TUNEL, in extended culture protocols to evaluate the activation status, cellular viability, and stromal function more comprehensively. Implementing these endpoints in future studies will be crucial to refine culture protocols and advance their potential for clinical application.

A major limitation of this study lies in the restricted availability of human ovarian cortical tissue, particularly from young cancer patients undergoing fertility preservation, which is extremely limited. This constraint, both ethical and methodological, limits the number of replicates and the range of assays that can be performed. For these reasons, morphological analysis was prioritized in the present study as an initial, yet informative, endpoint. Clinical validation of SSS in ovarian tissue transplantation is essential. Future studies should evaluate whether pre-culturing ovarian tissue with SSS improves follicle survival post-transplantation, potentially enhancing fertility preservation outcomes. By addressing these aspects, SSS could become a valuable supplement to reproductive medicine protocols, improving ovarian tissue viability and function.

## 5. Conclusions

This study demonstrates that supplementation with SSS, a serum substitute previously validated in IVF embryo culture, significantly enhances primordial follicles survival and maintains follicular morphology during short-term ovarian cortical tissue culture. By providing a more physiologically balanced environment, SSS may provide a more effective alternative to traditional supplements such as HSA and HS for early-stage follicle maintenance. These findings highlight the critical role of culture supplement composition in supporting ovarian tissue integrity and lay the basis for the development of fertility preservation strategies.

## Figures and Tables

**Table 1:** Patient characteristics and number of counted follicles.

Subject	Age (Years)	Pathology	Total Follicles Analyzed	Total PMFs Analyzed	Total PFs Analyzed	Total GFs Analyzed
1	14	Hemophagocytic syndrome	8244	4230	3888	126
2	15	Medulloblastoma	33,067	22,991	9943	133
3	25	Ewing’s sarcoma	24,675	16,997	7583	95

Clinical data of the three patients included in the study, detailing age, pathology, and the total number of follicles analyzed. Follicles were categorized into primordial follicles (PMFs), primary follicles (PFs), and growing follicles (GFs).

**Table 2:** Generalized linear mixed model (GLMM) analysis of follicle density predictors.

Predictors	Coefficient	Std. Error	*p*-Value	95% CI Lower	95% CI Upper
Treatment HSA	0.413	0.204	0.042	0.014	0.812
Treatment SSS	0.883	0.201	<0.001	0.490	1.276
Patient age	0.044	0.016	0.005	0.013	0.075
Intercept (cons)	−0.088	0.357	0.804	−0.788	0.611

Results of the GLMM evaluating the effects of culture conditions (HSA, HS, and SSS) and patient age on follicle density. Coefficients represent the estimated effect size for each variable, with standard errors (Std. Error), *p*-values, and 95% confidence intervals (CI) provided. Treatment with SSS was associated with a significant increase in follicle density compared to HS (*p* < 0.001), while HSA also showed a significant effect (*p* = 0.042). Patient age exhibited a significant positive correlation with follicle density (*p* = 0.005). The intercept represents the baseline follicle density.

## Data Availability

All data supporting the findings of this study are available within the article.

## Abbreviations

The following abbreviations are used in this manuscript:

OCFs	Ovarian Cortical Fragments
HSA	Human Serum Albumin
HS	Human Serum
SSS	Serum Substitute Supplement
PMFs	Primordial Follicles
POI	Primary Ovarian Insufficiency
OTC-T	Cryopreservation And Transplantation
BCM	Basic Culture Medium
GCs	Granulosa Cells
PFs	Primary Follicles
GFs	Growing Follicles
GLMM	Generalized Linear Mixed Model
IVF	In Vitro Fertilization
